# HumanFilt: a multi-reference host depletion pipeline improves *Fusobacterium* detection accuracy in tumor WGS data sets

**DOI:** 10.1128/msystems.00550-26

**Published:** 2026-07-14

**Authors:** Mariia Frolova, Barry Maguire, Heiko Duessmann, Menatallah Rayan, Caoimbhe Burke, Hesham M. Korashy, Arman Raman, William M. Gallagher, John P. Burke, Simon J. Furney, Jochen H. M. Prehn

**Affiliations:** 1 Department of Physiology and Medical Physics and RCSI Centre for Systems Medicine, Royal College of Surgeons in Ireland 8863, Dublin, Ireland; 2 Department of Colorectal Surgery, Beaumont Hospital 57978, Dublin, Ireland; 3 Department of Pharmaceutical Sciences, College of Pharmacy, QU Health, Qatar University 61780, Doha, Qatar; 4 UCD School of Medicine, UCD Conway Institute, University College Dublin 8797, Dublin, Ireland; Agroscope Standort Reckenholz, Zurich, Switzerland

**Keywords:** colorectal cancer, mucinous rectal adenocarcinoma, *Fusobacterium nucleatum*, *Fusobacterium animalis*, human-read filtering, immunohistochemical staining

## Abstract

The study of tumor-associated microbiomes using whole-genome sequencing (WGS) has attracted considerable attention, but microbial signal detection remains controversial due to host contamination and methodological artifacts. As the necessity of human-read removal becomes increasingly evident, many groups now include this step in their data pre-processing workflows. In this work, we introduce an open-source tool, HumanFilt, designed for rigorous host-read removal and apply it to the re-analysis of WGS data from 10 mucinous rectal adenocarcinoma cases originally published by Reynolds et al. The workflow integrates k-mer-based classification (Kraken2), quality and adapter trimming (Trim Galore), vector filtering (BBDuk/UniVec_Core), and duplicate removal (FastUniq). After reducing data complexity, a multi-aligner, multi-reference approach (BWA-MEM/GRCh38, Bowtie2/T2T-CHM13, and Minimap2/Human Pangenome Reference Consortium v1.1) removes remaining host sequences, collectively eliminating more than 99.9% of human-derived reads. Although the additional alignment steps eliminated only a small fraction of total reads, they consistently removed millions of residual sequences per sample, underscoring the importance of rigorous filtering in data sets where non-human reads are a small minority. Taxonomic profiling with PathSeq and MetaPhlAn revealed reproducible enrichment of *Fusobacterium* species in tumor versus matched normal tissues, and comparison before and after filtering showed that this tumor-over-normal pattern was preserved despite an overall reduction in RPM values. Simulation analyses further showed that HumanFilt preserved more than 99.7% of true *Fusobacterium* signals, supporting high specificity without meaningful false-negative loss of microbial reads. In direct comparison with Deacon and NoHuman, HumanFilt achieved the most stringent host-read removal but also removed a greater proportion of PathSeq-classified *Fusobacterium* reads, highlighting the trade-off between maximal host depletion and preservation of ambiguous microbial signal. Cross-validation with immunofluorescence analysis using pan-*Fusobacterium* (detecting both *Fusobacterium animalis* and *Fusobacterium nucleatum*) and *F. nucleatum*-specific antibodies showed general consistency with *Fusobacterium* subspecies detected by WGS. Compared to the unfiltered analysis, host depletion markedly reduced artificial microbial signals in normal samples while preserving tumor-associated *Fusobacterium*, resulting in a more reliable microbial profile.

**IMPORTANCE:**

We developed an open-source tool that enables rapid removal of human-derived sequences and applied it to rectal cancer whole-genome sequencing data. This approach reduced false microbial signals while preserving true tumor-associated *Fusobacterium*, and simulation analyses showed that it retained more than 99.7% of true *Fusobacterium* reads. Comparison with Deacon and NoHuman showed that HumanFilt achieved more stringent host depletion but also highlighted the trade-off between aggressive host filtering and preservation of ambiguous microbial signal. We also observed general consistency between the sequencing results and immunofluorescence staining in tissue. Together, these findings provide a more reliable basis for studying tumor-bacteria interactions.

## INTRODUCTION

The study of tumor microbiomes using whole-genome sequencing (WGS) has attracted significant attention as bacteria have been found in various types of solid tumors including colon, pancreatic, breast cancers (1, 2). These discoveries have sparked growing interest in the potential roles of bacteria in tumor development, progression, and response to therapy. However, the validity of microbial signals in tumor tissue remains debated.

One of the most widely publicized studies in this area, published by Poore et al. in 2020, claimed that microbial DNA can be detected in various tumor types using data from The Cancer Genome Atlas (3). However, the study was formally retracted (4) as independent investigations found serious methodological shortages. Re-analysis by Gihawi et al. (5) showed that the main cause of incorrect taxonomic assignments was insufficient removal of human reads. Moreover, bacterial reference genomes can contain human sequence insertions, leading to misclassification (6, 7). These findings have underscored to the research community the necessity of rigorous host read removal, and most recent studies now implement careful and comprehensive human-read filtering as a standard step (8, 9). Given these challenges, it is critical to apply strict quality control and human-read filtering before interpreting the presence of microbial sequences in tumor WGS data. Several host depletion tools have recently been developed for metagenomic applications, including NoHuman (10), Deacon (11), and Cleanifier (12). HumanFilt complements these tools by addressing the specific demands of tumor WGS data, where extreme human DNA fractions and tumor-associated genomic variation may reduce the effectiveness of single-reference, single-approach host filtering methods. The sequential multi-aligner design of HumanFilt is supported by Bush et al. (13), who demonstrated that combining two complementary aligners sequentially achieves superior human-read removal compared to any single-step approach. To validate computational results, it is important to compare WGS-based microbial signals with other techniques, such as immunodetection. While sequencing provides quantitative taxonomic resolution, immunostaining provides spatial and morphological context in addition to confirming these signals. Such cross-comparison is particularly important when studying closely related subspecies, where read misclassification and background noise may otherwise confound computational results.

A recent study demonstrated that *Fusobacterium animalis* is the predominant *Fusobacterium* subspecies detected in more colorectal tumor samples compared with *Fusobacterium nucleatum* (14). These findings highlight the importance of distinguishing between different *Fusobacterium* subspecies, as only *F. animalis* showed a prognostic impact, underscoring the need to separate their contributions to colorectal cancer development and progression.

Here we describe HumanFilt, a multi-reference host depletion pipeline developed for tumor WGS data and validated using simulated and clinical data sets. We further benchmark HumanFilt against existing host depletion tools and show that it preserves the tumor-over-normal *Fusobacterium* signal after rigorous filtering.

## MATERIALS AND METHODS

### Raw read analysis

We re-analyzed the WGS data set published by Reynolds (15), which includes 10 cases of mucinous rectal adenocarcinoma with matched tumor and adjacent normal tissues. Reads were processed with HumanFilt (v1.1.0), a workflow developed in-house and distributed via Bioconda, configured exclusively for paired-end Illumina data (https://github.com/jprehn-lab/humanfilt). The pipeline (i) pre-filters human reads with Kraken2 (16) against a curated database derived from GRCh38 (17) and T2T-CHM13 reference genomes; (ii) performs adapter and quality trimming with Trim Galore, discarding reads <20 bp and bases with Phred <20; (iii) collapses duplicate read pairs with FastUniq (18); (iv) removes sequencing-vector contamination using BBMap bbduk.sh (19) against the UniVec_Core database (August 2024 release; *k* = 27, hdist = 1); and (v) applies stepwise host-alignment filtering, retaining only pairs unmapped at each stage: first to GRCh38 with BWA-MEM (20), then to T2T-CHM13 (21) with Bowtie2 (22), and finally to the Human Pangenome Reference Consortium (HPRC) (v1.1) pangenome (23) with Minimap2 (24). HumanFilt optionally retains all intermediate FASTQ files at each processing step, enabling read-count verification and stepwise quality control throughout the pipeline. On first use, HumanFilt (v1.0.0) automatically downloads and caches all required references and databases from our Zenodo repository (https://doi.org/10.5281/zenodo.17020481), including GRCh38, T2T-CHM13, HPRC (v1.1), UniVec_Core, and the curated human Kraken2 database. All tools were obtained through Conda (Bioconda and conda-forge). The principal tools and their versions are listed in Table S0.

### Taxonomic analysis using PathSeq and MetaPhlAn

Filtered non-human reads were subjected to taxonomic profiling with two complementary methods. PathSeq (GATK v4.6.1.0) (25) was run against a curated *Fusobacterium* reference database that had been pre-screened to exclude potential human insertions using a sliding-window strategy (150 bp windows, 75 bp steps) derived from GRCh38, T2T-CHM13 (v2.0), and HPRC (v1.1), aligned against *Fusobacterium* genomes with Bowtie2. PathSeq classification was performed with internal host-read filtering enabled throughout (GRCh38 reference), and only unambiguous *Fusobacterium* reads were counted to ensure consistent assessment across filtering conditions. In parallel, MetaPhlAn (mpa_vJan25_CHOCOPhlAnSGB_202503) (26) was applied with default parameters to provide independent estimates of microbial proportional abundance. Proportional abundances of *Fusobacterium* genus members and selected clades (*F. nucleatum* and *F. animalis*) were quantified in tumor and matched normal samples. Results were compared before and after HumanFilt processing to assess the impact of stricter human-read filtering on microbial signal detection.

To benchmark the performance of HumanFilt under controlled conditions, simulated paired-end Illumina reads were generated using ART (ART_Illumina v2.5.8) (27) with the HiSeq 2500 error profile (--ss HS25). Reads of 150 bp were simulated with a mean insert size of 350 bp (±50 bp SD) and a fixed random seed (--rs 42) to ensure reproducibility. *Fusobacterium nucleatum* subsp. *nucleatum* strain ATCC 25586 (NCBI accession number NZ_CP028101.1) was simulated at 1,000× coverage. Human genomic reads were independently simulated at 30× coverage from three reference assemblies: GRCh38, T2T-CHM13v2.0, and the HPRC reference.

Mixed data sets mimicking tumor-derived sequencing data with known microbial content were constructed using SeqKit (28) and custom bash scripting. For each human reference, 10,000,000 read pairs were subsampled from the simulated human reads and combined with 100,000 *F. nucleatum* read pairs, yielding an approximate microbial fraction of ~1% per mixed data set. This resulted in seven data sets in total: three human-only controls (GRCh38, T2T, and HPRC), one *F. nucleatum*-only control, and three mixed data sets (GRCh38 + Fuso, T2T + Fuso, and HPRC + Fuso). All data sets were processed through HumanFilt and compared against alternative host depletion tools to evaluate the sensitivity and specificity of microbial read retention.

### Histological slice preparation, staining, and image analysis

Formalin-fixed paraffin-embedded (FFPE) tumor sections from primary tumor sections of mucinous rectal cancer patients following resection at the Beaumont RCSI Cancer Center were obtained. Sections were cut (5 µm), baked, and mounted on individual slides. Prior to staining, the slides were baked at 60°C overnight. The Leica BOND RXm platform (Leica Biosystems, Nussloch, Germany) was used henceforth for slide preparation and staining. Sections underwent deparaffinization, rehydration, permeabilization in 0.3% Triton X-100 in PBS for 10 min, and heat-mediated epitope retrieval using Bond ER1 (citrate based, pH 6.0) and ER2 (Tris based, pH 9.0) buffers. Slides were then incubated in blocking solution (10% donkey serum and 3% BSA in PBS) for 1 h at room temperature. After blocking, the slides were immersed in PBS and exposed to a multi-spectrum LED for 48 h to photoirradiate the tissue. Nuclei were stained with DAPI 1 µg/mL for 15 min, and the Cell DIVE platform (Leica Microsystems, Wetzlar, Germany) was used to perform 2× whole-slide imaging and tissue selection, 10× whole-tissue imaging and region selection, and 20× autofluorescence imaging. Following that, during the first round of staining, slides were incubated for 1 h with a commercial antibody (ANT0084 1:100; Diatheva, Cartoceto, Italy) raised against *F. nucleatum* subsp. *nucleatum* ATCC 25586, which preferentially detects *F. nucleatum*. Following primary antibody staining, slides were washed three times for 30 s in BOND Wash buffer and then incubated for 1 h at room temperature with the Alexa Fluor 647 donkey anti-rabbit antibody (Abcam, Cambridge, UK). Subsequently, the slides were stained using a pan-*Fusobacterium* antisera (1:100) raised against purified total membrane proteins from 11 *Fusobacterium* strains (*F. nucleatum* subsp. *nucleatum* ATCC 23726, *F. nucleatum* subsp. *animalis* ATCC 7_1, *F. nucleatum* subsp. *polymorphum* ATCC 10953, *F. nucleatum* subsp. *vincentii* ATCC 49256, *F. periodonticum* ATCC 2_1_31, *F. varium* ATCC 27725, *F. ulcerans* ATCC 49185, *F. mortiferum* ATCC 9817, *F. gonidiaformans* ATCC 25563, *F. necrophorum* subsp. *necrophorum* ATCC 25286, and *F. necrophorum* subsp. *funduliforme* ATCC 1_1_36S) (29), recognizing both *F. nucleatum* and *F. animalis* (30, 31), followed by an Alexa Fluor 555 donkey anti-rabbit antibody (Abcam). Nuclei were counterstained with DAPI, and 20× biomarker imaging was undertaken between each staining round. Digital images were processed and analyzed using QuPath (v0.6.0) for tissue annotation, background correction, and visualization. To confirm the appearance of *Fusobacterium* morphology, slides were then coverslipped and imaged using the LSM 980 Airyscan 2 confocal microscope (Carl Zeiss, UK) using a 63 × 1.4 NA oil immersion objective. Confocal z-stacks of optical slices of *Fusobacterium*-positive regions were at 150 nm intervals with a pixel size of 35 × 35 nm using the 4Y-mode. Images were processed with ZEN software (Carl Zeiss).

### Cell and bacterial co-culture, and staining and imaging

*Fusobacterium* was cultured overnight in fastidious anaerobe broth (FAB, Neogen) at 37°C under 0.1% O_2_ using an anaerobic chamber (Whitley A35, Don Whitley Scientific). To remove dissolved oxygen, FAB and PBS were autoclaved and allowed to equilibrate overnight in the anaerobic chamber prior to use. Cancer cells (MDA-MB-468) were seeded in cover glass-bottom cell culture dishes (m-dish #81156; ibidi, Germany). Once desired confluency was reached (70%–80%), cells were infected with an optimized multiplicity of infection of 1:125. After 12 h of co-culture, extracellular bacteria were removed by washing twice with PBS and subsequent incubation for 1 h in DMEM/F-12 containing 200 mg/mL metronidazole and 300 mg/mL gentamicin. Metronidazole and gentamicin were selected for their complementary mechanisms of action against *Fusobacterium*: metronidazole targets anaerobic bacteria, while gentamicin eliminates residual extracellular gram-negative organisms, consistent with previously published co-culture protocols (32). Cells were then washed and fixed with 4% formalin for 20 min at RT and stored at 4°C.

Immunocytochemical (ICC) staining of cells was undertaken. First, cells were permeabilized using a buffer containing 1% BSA, 2% horse serum, and 0.1% Triton in PBS for 30 min at room temperature. Cells were washed three times with PBS for 3 min each. Antibody solutions were prepared in the ICC buffer with either the commercial *F. nucleatum* antibody (1:100) (ANT0084) or the pan-*Fusobacterium* antisera (1:100), along with a pancytokeratin antibody (1:100) (Abcam) and incubated for 1 h at room temperature. After three further 3-min washes with PBS, cells were stained with a cocktail solution of Alexa Fluor 555 goat antirabbit antibody (1:1,000) (Abcam) and Alexa Fluor 647 donkey antimouse antibody (1:1,000) (Abcam) for 1 h at room temperature. Following a subsequent wash, cells were incubated with Hoechst (1 mg/mL) for 30 min at room temperature. After a final round of PBS washes, cells were imaged on a confocal super-resolution microscope (LSM 980 Airyscan 2, Carl Zeiss) using a 63 × 1.4 NA oil immersion objective. Stacks of optical slices were taken at an interval of 150 nm with a pixel size of 35 nm × 35 nm using the 4Y-mode. Imaging data were prepared for visualization using the same range of the histogram for each staining in ZEN (Carl Zeiss).

## RESULTS

### Filtering of human reads

**Fig 1:**
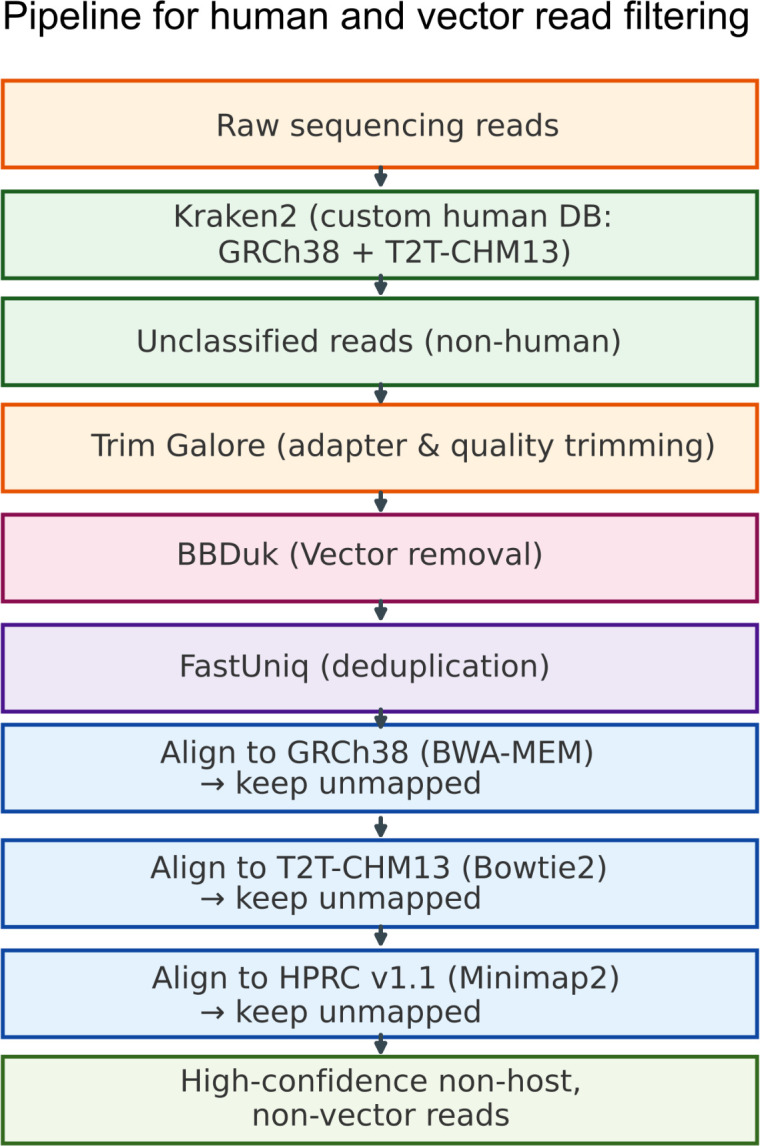
Workflow for filtering human and vector reads from WGS data.

We implemented a stepwise pipeline to remove host and contaminant sequences from mucinous rectal cancer WGS data (Fig. 1). The workflow HumanFilt-1.1.0 removed human and vector sequences, with read counts recorded at each processing step and compiled into a final summary report for each sample (https://github.com/jprehn-lab/humanfilt). The same 10 matched tumor and normal tissue samples (Cases A–J) are referred to consistently by the same identifiers throughout the Results section, figures, and Tables S1 to S11. Raw reads were first processed with Kraken2 (16) against a curated human database (GRCh38 + T2T-CHM13), removing 99.6% ± 0.08% of total reads per sample. This database was used to maximize early removal of host-derived reads while keeping the pre-screening step fast and computationally inexpensive. Because Kraken2 is k-mer-based, adapter sequence has negligible impact at this stage, so this pass both accelerates processing and significantly reduces the data set for subsequent steps. We then applied Trim Galore (33) (Phred ≥20, minimum length 20 bp) for adapter and quality trimming to improve mapping ability, followed by FastUniq (18) to collapse duplicate read pairs that could otherwise inflate downstream read-count estimates. Sequencing-vector contamination was filtered with BBDuk (BBTools v37.62) (19) against UniVec_Core (August 2024; *k* = 27, hdist = 1) to remove residual technical/vector-derived sequences before alignment-based host depletion, typically removing ~54,000 to 223,000 reads per sample. Finally, the remaining unclassified reads were aligned sequentially to GRCh38 (17) (BWA-MEM [20]), T2T-CHM13 (21) (Bowtie2 [22]), and the HPRC (v1.1) pangenome (23) (Minimap2 [24]), retaining only pairs unmapped at each stage. These alignment filters removed an additional 0.23%–0.38% of residual human reads ~1.7 to 4.0 million per sample, yielding the final high-confidence non-host set. Read counts at each stage of HumanFilt were computed with SeqKit (28). The proportion of reads retained after each filtering step is summarized in Table 1 (normalized), Table S1 (not-normalized, tumor samples), and Table S2 (not-normalized, normal samples).

**TABLE 1:** Read processing summary for mucinous rectal cancer WGS data

Case ID	Total reads (M)	Kraken2 unclassified (T)		Trimmed reads (T)		Vector and duplicate reads removed (T)		Unaligned reads (GRCh38 + T2T + HPRC) (T)	
A	1,010	4,484, 0.44%		4,422, 0.44%		4,328, 0.43%		815, 0.08%	
B	610	1,900, 0.31%		1,853, 0.3%		1,799, 0.29%		146, 0.02%	
C	1,013	2,832, 0.28%		2,759, 0.27%		2,680, 0.26%		295, 0.03%	
D	1,011	4,583, 0.45%		4,516, 0.45%		4,423, 0.44%		944, 0.09%	
E	1,007	3,083, 0.31%		3,021, 0.3%		2,921 0.29%		252, 0.02%	
F	938	2,738, 0.29%		2,655, 0.28%		2,567, 0.27%		209, 0.02%	
G	1,015	3,577, 0.35%		3,491, 0.34%		3,374, 0.33%		542, 0.05%	
H	1,022	3,971, 0.39%		3,783, 0.37%		3,597, 0.35%		853, 0.08%	
I	1,092	4,945, 0.45%		4,757, 0.44%		4,534, 0.41%		967, 0.09%	
J	551	2,684, 0.49%		2,613, 0.47%		2,512, 0.46%		455, 0.08%	

### Comparison of *Fusobacterium* and *F. nucleatum* normalized read counts before and after human-read filtering by HumanFilt

We assessed the impact of each HumanFilt filtering step on *Fusobacterium* detection by comparing PathSeq-derived RPM values at each pipeline stage, identifying at which step the greatest read loss occurred. RPM values were calculated as reads per million unambiguous *Fusobacterium* reads at both genus and species levels across all 10 matched tumor and normal samples.

Before constructing the PathSeq (25) *Fusobacterium* database, we assessed all reference genomes included in the genus for potential human sequence insertions. Using the sliding-window strategy described by Sepich-Poore et al. (34), 150 bp sequences were generated at 75 bp intervals from GRCh38, T2T-CHM13v2.0, and the HPRC contig assembly (v1.1), excluding fragments shorter than 75 bp or containing more than 100 ambiguous bases. These sequences were aligned to the *Fusobacterium* reference genomes using Bowtie2 (22). No human-derived sequences aligned to any *Fusobacterium* genome, confirming that the genus-wide references are free from human DNA contamination.

**Fig 2:**
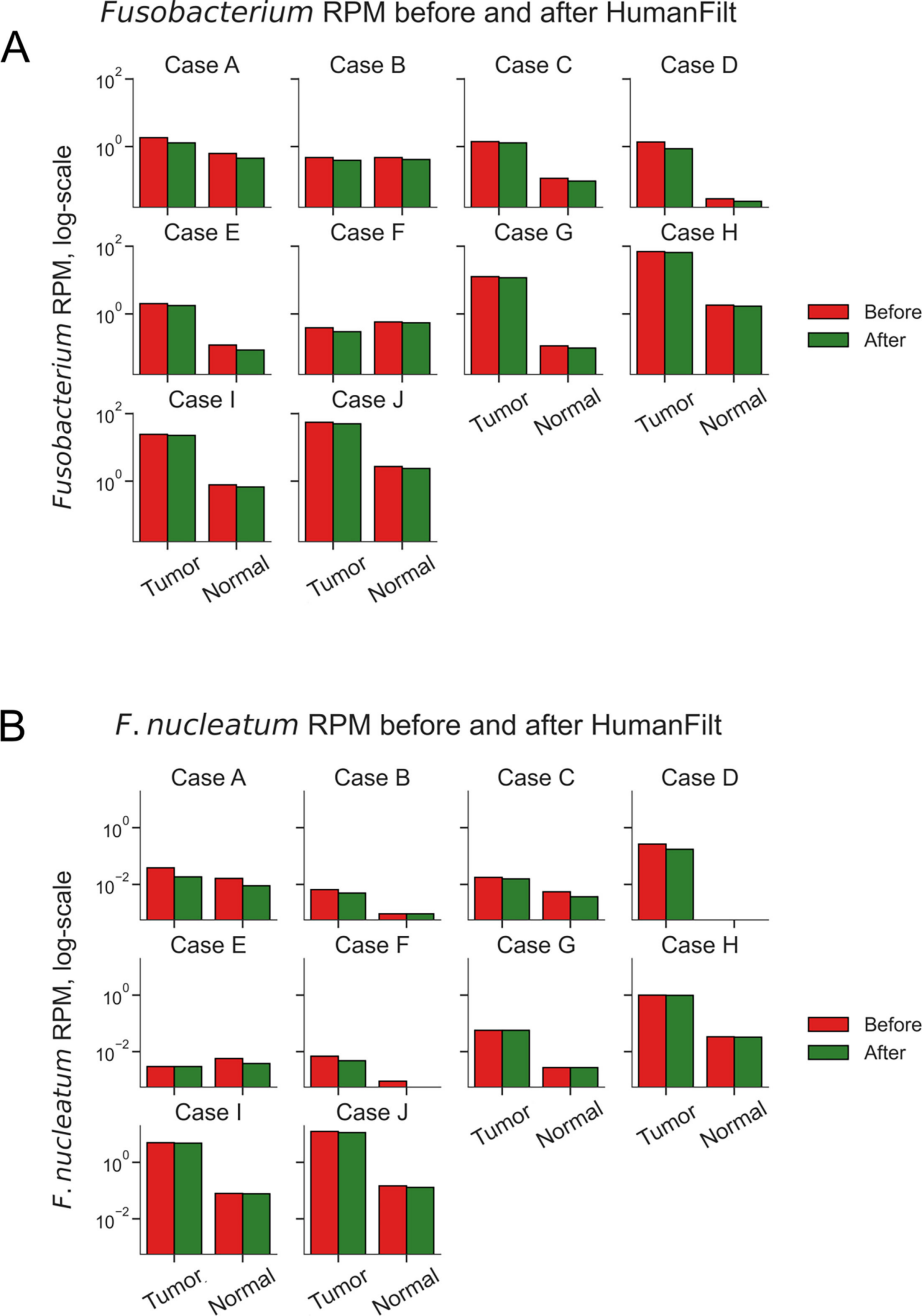
Normalized read abundance(reads per million [RPM]) of *Fusobacterium* in matched normal and tumour tissue samples from patients with mucinous adenocarcinoma of the rectum: (**A**) *Fusobacterium* genus and (**B**) *Fusobacterium nucleatum* species, before and after humanread depletion with HumanFilt.

To assess the impact of host read removal on *Fusobacterium* genus detection, we compared RPM values before and after HumanFilt processing across 10 matched tumor and normal tissue pairs (Cases A–J) (Fig. 2A; Table S2). *Fusobacterium* was detected by PathSeq in both conditions, with tumor samples consistently showing higher RPM counts than matched normal tissues (Fig. 2A). Overall, HumanFilt removed a mean of 16.0% of PathSeq-classified *Fusobacterium* reads across tumor samples (range: 6.44%–36.06%), with the majority of this loss attributable to alignment against the GRCh38 human reference genome (BWA_GRCh38), which alone accounted for a mean loss of 13.07% (range: 5.47%–25.7%). The preceding quality control steps—Kraken2 (mean 1.77%), TrimGalore (0.12%), Dedup (0.40%), and BBDuk (0.61%)—contributed only marginal reductions, while the subsequent alignment steps against the T2T-CHM13 genome (Bowtie2_T2T) and the Human Pangenome Reference Consortium assembly (Minimap2_HPRC) introduced negligible additional loss (<0.03%). These findings indicate that BWA_GRCh38 alignment is the primary stage at which somatically mutated human reads, misclassified as *Fusobacterium* by PathSeq, are correctly identified and removed. The largest reductions were observed in Case A (−30.5% tumor, −26.9% normal), Case D (−36.1% tumor, −15.6% normal), and Case E (−28.6% normal), likely reflecting higher proportions of ambiguous reads in these samples. By contrast, cases with high RPM values, such as Cases G, H, I, and J, showed minimal impact after filtering (6.4%–10.2%), suggesting that the *Fusobacterium* signal in these samples is robust and predominantly of true microbial origin. Overall, the tumor-over-normal enrichment pattern was preserved across the majority of cases, supporting the biological specificity of *Fusobacterium* detection within our pipeline.

At the species level, *F. nucleatum* RPM values before and after HumanFilt processing showed greater variability than observed at the genus level (Fig. 2B, Table S4). In tumor samples, the median RPM decrease was 16.6% (range: 0.0%–52.6%), with Case A (−52.6%) and Case D (−34.5%) showing the largest reductions, consistent with low normalized *Fusobacterium* read counts making these reads more susceptible to competitive alignment during host removal. Cases E and G showed no change (+0.0%), while cases with high RPM values, such as Cases H, I, and J, showed minimal impact (−0.9%, −3.4%, and −7.5%, respectively), suggesting that *F. nucleatum* signal in these samples is robust. In normal samples, the median RPM decrease was 25.4% (range: 0.0%–100.0%); the apparent 100% reduction in Case F normal sample reflects near-zero baseline normalized read counts (0.0009 RPM before filtering) rather than a biologically meaningful loss. Case D normal showed 0 RPM both before and after filtering, indicating absence of detectable *F. nucleatum* in matched normal tissue. Overall, the sparse and heterogeneous distribution of *F. nucleatum* across cases accounts for the wider variability at the species level compared to genus-level patterns.

Detailed counts and RPM values for *Fusobacterium* and *F. nucleatum* for all samples are provided in Tables S3 and S4, respectively.

### Validation of HumanFilt sensitivity and specificity using simulated data sets

To evaluate the capacity of HumanFilt to preserve true microbial signal while eliminating host reads, we constructed three classes of simulated data sets and processed them through the complete pipeline, recording total and *Fusobacterium*-specific read counts at each step (Table S5, total read counts; Table S6, PathSeq *Fusobacterium* counts).

First, to assess false-negative risk—that is, unintended removal of microbial reads—a data set consisting solely of 7,267,000 simulated *F. nucleatum* read pairs (strain ATCC 25586, NZ_CP028101.1) was processed through HumanFilt. Stepwise filtering through Kraken2, TrimGalore, deduplication, and BBDuk resulted in only marginal total read losses (99.70% retained after pre-alignment steps), and sequential alignment against GRCh38, T2T-CHM13v2.0, and HPRC references reduced the final output to 6,973,727 read pairs (95.96% of input) (Table S5). Critically, PathSeq-detected unambiguous *Fusobacterium* read counts increased from 7,570,471 prior to host depletion to 7,773,976 following the complete pipeline, yielding a net retention rate of 102.69% (Table S6). This modest increase reflects the fact that, in the absence of competing human reads, previously ambiguous *Fusobacterium* reads are resolved into unambiguous classifications by PathSeq, confirming that HumanFilt introduces no false-negative losses for microbial signal.

Second, to evaluate pipeline specificity, we processed three human-only data sets—derived from GRCh38 (313,643,361 read pairs), T2T-CHM13v2.0 (311,727,375 read pairs), and HPRC (301,588,386 read pairs)—through the complete HumanFilt workflow. In all cases, Kraken2 reduced total read counts by >99.8% in a single step, with residual reads further eliminated by sequential alignment (Table S5). Final outputs were zero read pair for GRCh38- and HPRC-derived data sets (100% removal) and one residual read pair (two reads) for the T2T-derived data set (>99.9999% removal) (Table S5). Notably, in the HPRC-derived data set, 53,455 read pairs (0.018% of the original input, 94.2% of post-GRCh38 residual reads) survived both GRCh38 and T2T-CHM13v2.0 alignment and were exclusively eliminated at the final Minimap2 HPRC pangenome step. This demonstrates that a substantial fraction of human reads—representing genomic regions absent or insufficiently represented in both GRCh38 and T2T-CHM13v2.0—are uniquely captured by the HPRC pangenome reference, directly justifying the sequential multi-reference design of HumanFilt. PathSeq detected zero *Fusobacterium* reads at every processing stage across all three human-only data sets (Table S6).

Third, to simulate realistic clinical WGS conditions, we constructed three mixed data sets each comprising 10,000,000 human-read pairs derived from GRCh38, T2T-CHM13v2.0, or HPRC reference genomes, respectively, spiked with 100,000 simulated *F. nucleatum* read pairs (strain ATCC 25586, NZ_CP028101.1), yielding a human-to-microbial ratio of 100:1. In all three mixtures, Kraken2 performed the dominant host depletion in a single step, reducing total read pairs from 10,100,000 to 119,077 (GRCh38 derived, 98.82% removed), 131,634 (T2T derived, 98.70% removed), and 142,026 (HPRC derived, 98.59% removed) (Table S5). Subsequent alignment-based filtering steps further refined each data set, yielding final outputs of 99,670, 99,402, and 99,663 read pairs for the GRCh38-, T2T-, and HPRC-derived mixtures, respectively. Critically, PathSeq-detected *Fusobacterium* read counts remained stable throughout the entire pipeline across all three mixed data sets, with final values of 142,873 (GRCh38 mixture, 99.92% retention relative to pre-depletion baseline), 142,866 (T2T mixture, 99.91%), and 142,583 (HPRC mixture, 99.71%) (Table S6). These results confirm that HumanFilt effectively removes the overwhelming majority of human reads while preserving true microbial signal.

### Comparison of host depletion tools in tumor samples

To contextualize HumanFilt within the landscape of existing host depletion methodologies, we performed a direct comparison against two published host removal tools: Deacon and NoHuman. The sequential use of complementary alignment-based methods for host read removal was recommended by Bush et al. (13), who demonstrated that combining Bowtie2 and SNAP sequentially maximizes human-read removal while minimizing microbial read loss, a principle underlying HumanFilt’s multi-step design. Cleanifier, a further pangenome-aware tool employing gapped k-mer classification (12), was not benchmarked as it operates on the same underlying principle as Deacon and NoHuman.

**Fig 3:**
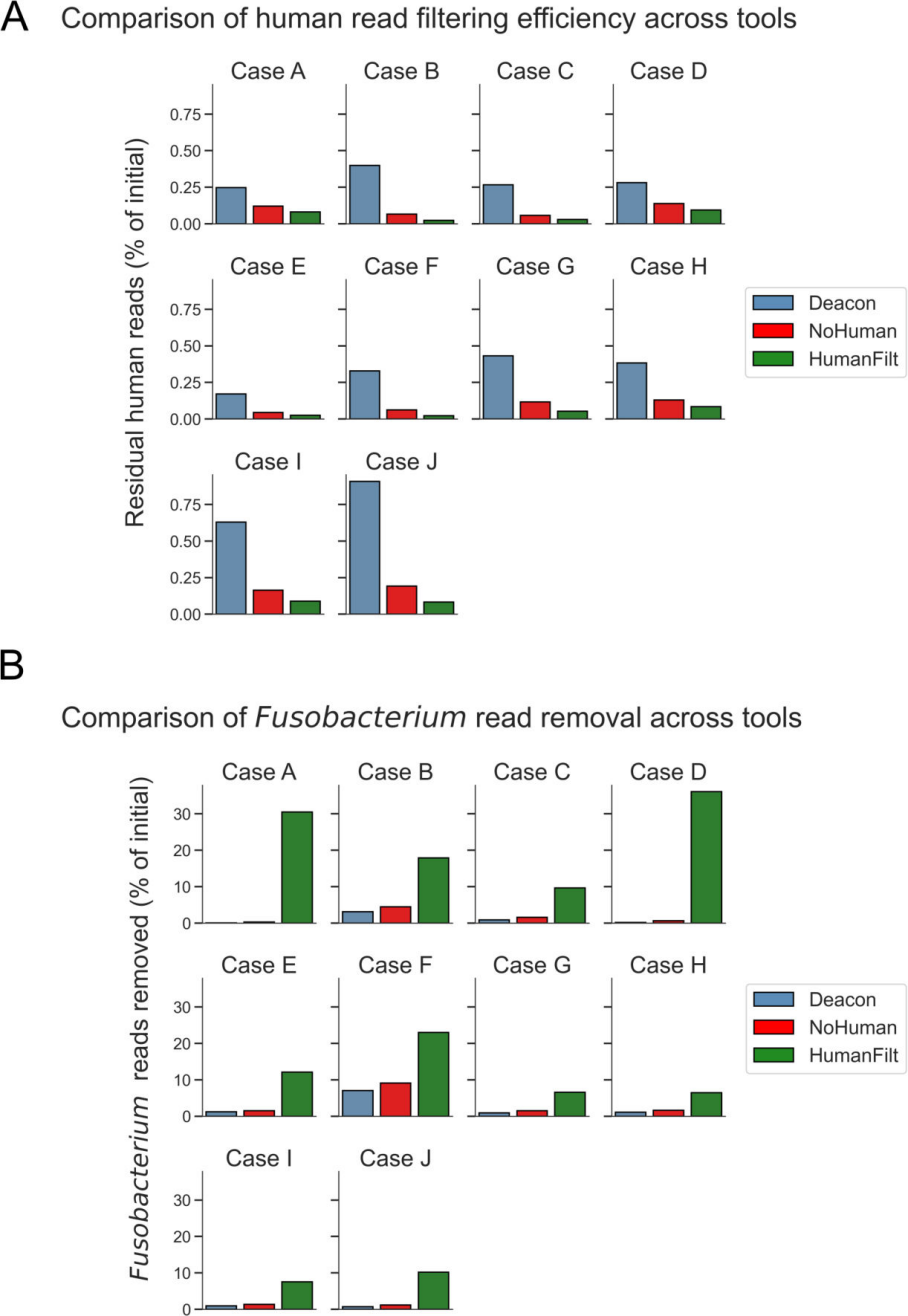
Performance of host depletion tools in matched normal and tumor tissue samples from patients with mucinous adenocarcinoma of the rectum. (**A**) Comparison of human-read filtering efficiency across host depletion tools. Percentage of human reads removed by HumanFilt, Deacon, and NoHuman. (**B**) Comparison of *Fusobacterium* read loss across host depletion tools. Percentage of *Fusobacterium* reads removed by HumanFilt, Deacon, and NoHuman.

HumanFilt achieved the most stringent host read removal across all tumor samples, retaining 0.02%–0.09% of initial reads after filtering, compared to 0.17%–0.91% for Deacon and 0.04%–0.19% for NoHuman (Table S7; Fig. 3A). These results confirm that HumanFilt’s sequential multi-aligner strategy removes a substantially greater proportion of human-derived reads than single-step k-mer-based host removal tools.

To evaluate tool specificity, we tracked *Fusobacterium* read counts before and after host depletion across all tumor samples (Table S8; Fig. 3B). Deacon and NoHuman preserved the vast majority of *Fusobacterium* reads, with mean off-target removal rates of 1.6% (range: 0.08%–7.02%) and 2.3% (range: 0.29%–9.08%), respectively. In contrast, HumanFilt removed a higher proportion of PathSeq-classified *Fusobacterium* reads, with a mean loss of 16% (range: 6.44%–36.06%). Simulation controls demonstrated that HumanFilt does not remove true *Fusobacterium* reads but instead improves their classification accuracy. The observed difference therefore likely arises from somatically mutated human reads misclassified as *Fusobacterium* by PathSeq, which were incorrectly retained by Deacon and NoHuman.

### Correlation between WGS-based clade detection and α-*Fusobacterium* immunostaining

**Fig 4:**
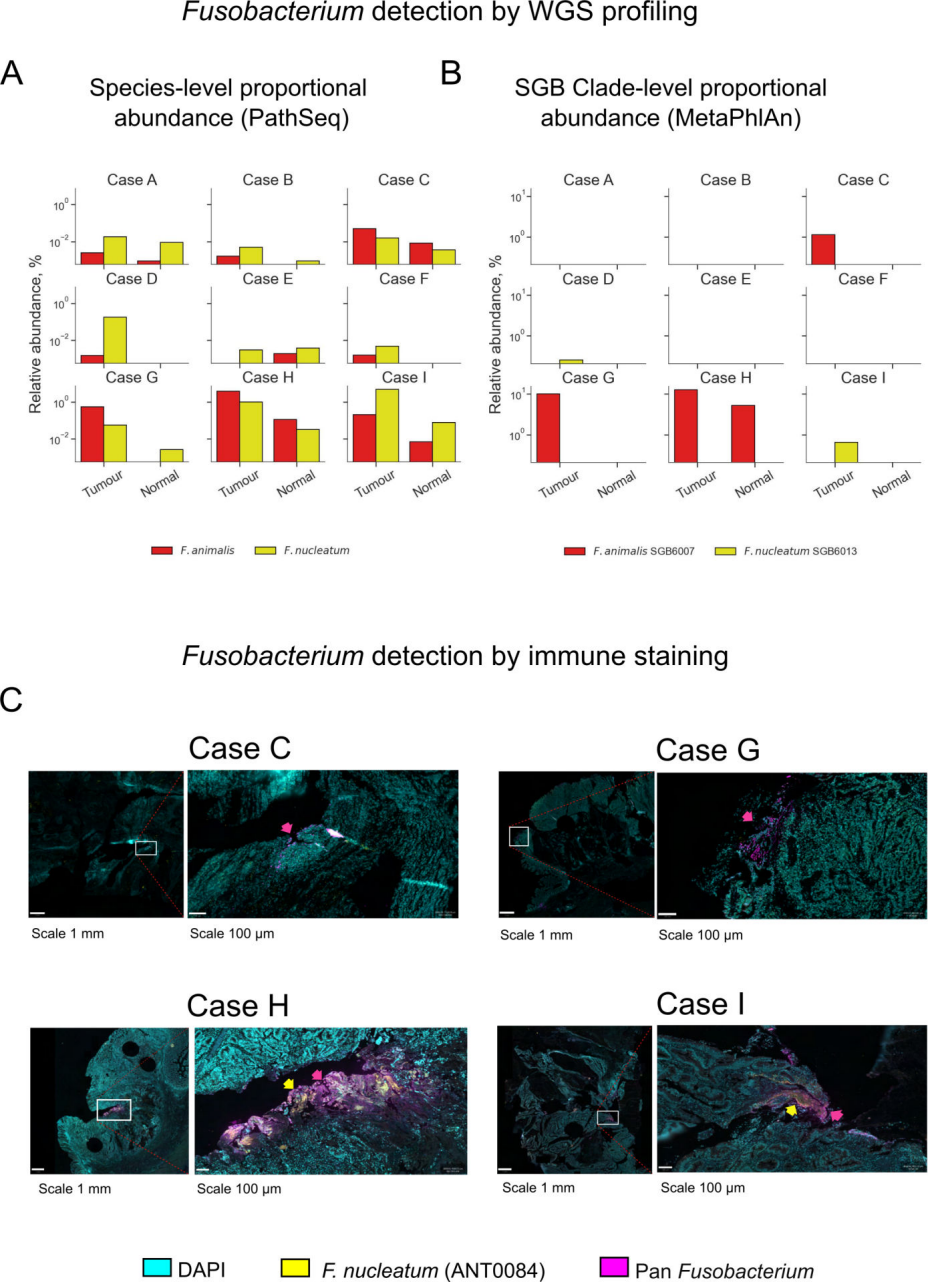
Detection of *F. nucleatum* and *F. animalis* by sequencing and immunostaining. (**A**) Proportional abundance (%, log scale) of *F. nucleatum* and *F. animalis* across tumour and normal samples as determined by PathSeq. (**B**) Proportional abundance (%, log scale) of the same species as determined by MetaPhlAn, where *F. animalis* was identified as SGB6007 and *F. nucleatum* as the SGB6013 clade. (**C**) Immunostaining results using two rabbit-derived *Fusobacterium* antibodies: the pan-*Fusobacterium* antibody (detecting both *F. nucleatum* and *F. animalis*) and the commercial *F. nucleatum*-specific ANT0084 antibody.

To determine whether WGS-based detection of *Fusobacterium* reflects bacterial presence within tumor tissue, we compared the PathSeq (25) and MetaPhlAn (26) outputs with immunostaining using the pan-*Fusobacterium* and *F. nucleatum*-specific (ANT0084) antibodies (Fig. 4). PathSeq was used as the primary read-based approach to quantify unambiguous *Fusobacterium* reads as RPM after host filtering. MetaPhlAn was included as an independent, marker-gene-based profiling method to assess whether the same dominant *Fusobacterium* clades were recovered using a different taxonomic strategy. Agreement between PathSeq, MetaPhlAn, and immunostaining was therefore used as supportive evidence for the presence and spatial localisation of *Fusobacterium* in tumor tissue, rather than as a definitive quantitative validation.

Immunohistochemical staining was performed on four cases (Cases C, G, I, and H) selected on the basis of tissue availability. In the stained samples, MetaPhlAn detected only *F. animalis* (SGB6007) and *F. nucleatum* (SGB6013), while other *Fusobacterium* clades (SGB6011, SGB6001, SGB6014, SGB5985, and SGB5986) were not identified (Table S10). This is relevant because the pan-*Fusobacterium* antibody can detect multiple *Fusobacterium* species. Although low-level reads from other species were detectable by PathSeq (Table S7), they were not dominant and are unlikely to explain the observed staining patterns with the pan-*Fusobacterium* antibody. Thus, staining only with the pan-*Fusobacterium* antibody suggests the presence of *F. animalis*; staining only with ANT0084 indicates *F. nucleatum*; and positive staining with both antibodies supports the presence of both species.

Case C and Case H both provide strong examples of *F. animalis*-positive, *F. nucleatum*-low tissue as validated by both WGS and immunostaining. In these cases, species quantification through WGS showed clear dominance of *F. animalis*, while *F. nucleatum* was detected only at background or very low levels (Table 2; Tables S9 and S10). Immunostaining results corresponded, showing positive staining with pan-*Fusobacterium* antibody but little to no staining with the *F. nucleatum*-specific antibody (Fig. 4C). This pattern suggests that while small counts of *F. nucleatum* may be present, they are below the threshold for antibody detectability and unlikely to represent a major component of the microbial community in these samples.

**TABLE 2:** Comparison of WGS-based *Fusobacterium* detection and immunostaining results

Case ID	Dominant species by WGS	PathSeq (RPM)	MetaPhlAn (%)	Pan-*Fusobacterium* staining^*a*^	*F. nucleatum*-specific (ANT0084) staining^*a*^	Interpretation
Case C	*F. animalis*	0.05	1.13	+	–	*F. animalis*
Case G	*F. animalis*	Fa 1.6/Fn 0.13	Fa 10.2/Fn 0	+	+	*F. animalis* predominant
Case I	*F. nucleatum*	Fa 0.2/Fn 4.8	Fa 0/Fn 0.65	+	+	*F. nucleatum* predominant
Case H	*F. animalis*	3.9	12.7	+	–	*F. animalis*

^*a*^ +, positive immunostaining; −, negative immunostaining.

For the *F. nucleatum-positive* category, Case I serves as an example, showing high levels of *F. nucleatum* by WGS (Table 2; Tables S9 and S10) and strong staining with both the *F. nucleatum*-specific and pan-*Fusobacterium* antibodies (Fig. 4), indicating *F. nucleatum* predominance within the tissue sections. Although the pan-*Fusobacterium* antibody can also detect *F. animalis*, the absence (or only trace levels) of *F. animalis* reads in the WGS data supports that the observed staining primarily reflects *F. nucleatum*.

In contrast, Case G showed high *F. animalis* RPM values and a low but detectable *F. nucleatum* signal by WGS (Table 2; Table S9). Both the pan-*Fusobacterium* and *F. nucleatum*-specific antibodies exhibited positive immunostaining (Fig. 4); however, the WGS data indicated a predominance of *F. animalis*, suggesting that the tissue was primarily colonized by this species.

Overall, we observed a general consistency between WGS-based *Fusobacterium* detection and antibody staining patterns in all examined cases. (Table 2; Fig. 3).

### Validation of species-specific immunostaining using controlled infections

To validate species-specific immunostaining, commercial strains of *F. nucleatum* (ATCC 25586) and *F. animalis* (ATCC 51191) were separately co-cultured with MDA-MB-468 breast cancer cells. Infected and control cells were stained with the pan-*Fusobacterium* antiserum and a commercial *F. nucleatum-*specific antibody (ANT0084), alongside pancytokeratin and Hoechst nuclear stain. High-resolution confocal microscopy (Zeiss LSM 980 Airyscan 2) was employed to acquire optical stacks.

**Fig 5:**
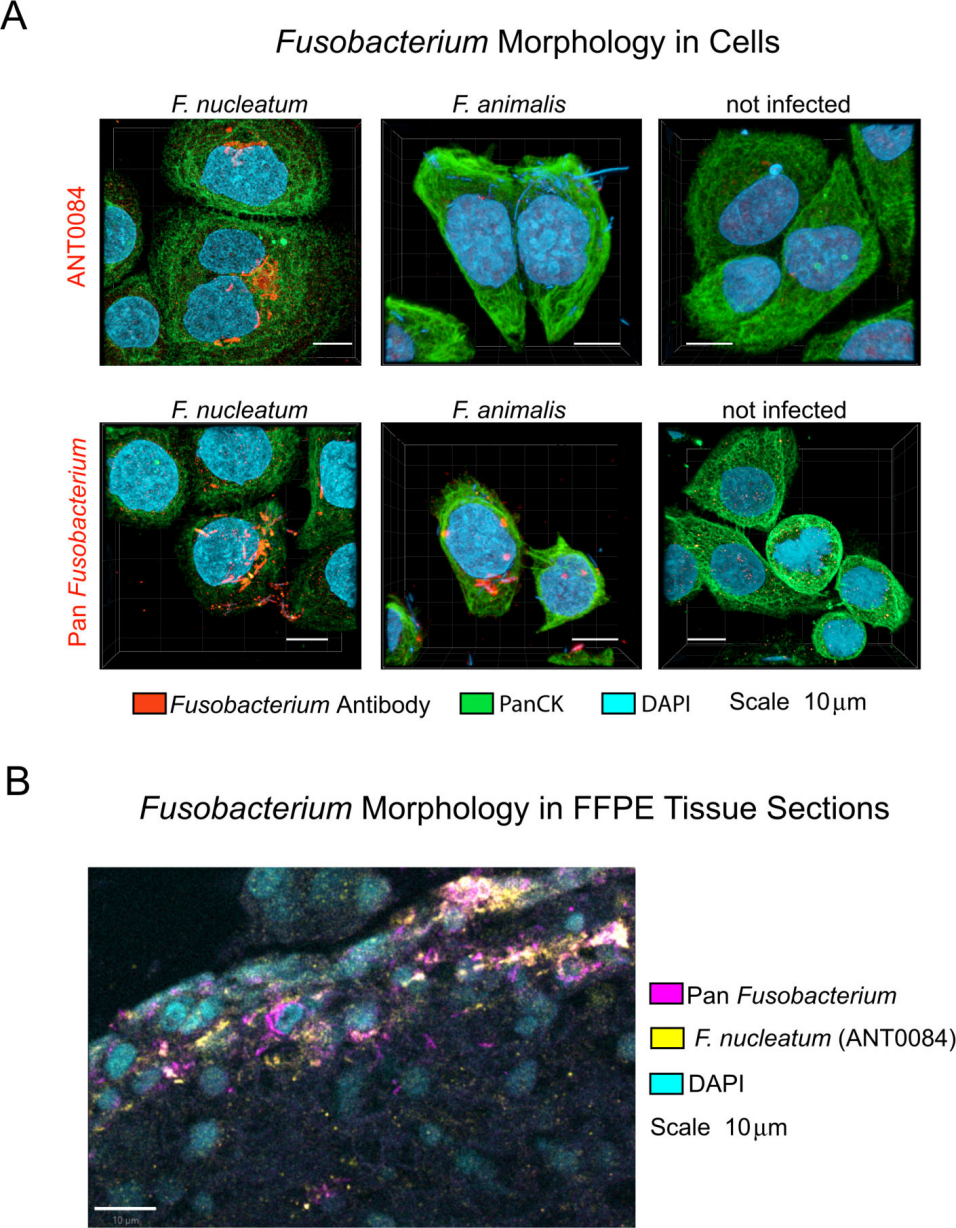
Immunofluorescence validation of *Fusobacterium* in MDA-MB-468 cells. (**A**) Cells were infected with *F. nucleatum* (left column) and *F. animalis* (middle column) and not infected (right column). They were stained with the commercially available ANT0084 (top row) and pan-*Fusobacterium* (**B**) Shown are maximum intensity projections of z-stacks; the scale bar is 10 mm in *x* and *y*. Imaging parameters: Zeiss LSM 980 Airyscan 2, 63×/1.4 NA; voxel size 35 × 35 × 150 nm and dz); estimated resolution Δ*x* = Δ*y* ≈ 120 nm, Δ*z* ≈ 400 nm; field of view (X) ≈ 44.47 µm. Images acquired as optical section stacks.

Cells infected with *F. animalis* displayed clear intracellular fluorescence with the pan-*Fusobacterium* antiserum (Fig. 5A, bottom middle) but no staining with ANT0084 (Fig. 5A, top middle), confirming that the pan-*Fusobacterium* reagent detects *F. animalis*, while ANT0084 is specific to *F. nucleatum*. Conversely, *F. nucleatum*-infected cells exhibited robust staining with both the pan-*Fusobacterium* antiserum (Fig. 5A, bottom left) and ANT0084 (Fig. 5A, top left). Uninfected control cells stained with ANT0084 were negative (Fig. 5A, top right), while those stained with the pan-*Fusobacterium* antiserum showed only very weak background signals (Fig. 5A, bottom right), indicating high specificity.

Taken together, these data confirm that the pan-*Fusobacterium* antiserum recognizes multiple *Fusobacterium* species, including *F. nucleatum* and F. *animalis*, whereas ANT0084 is a reliable species-specific antibody for *F. nucleatum*. This dual approach supports the WGS-based assignments of *Fusobacterium* species in tissue analyses.

To extend these findings to clinical material, we included a representative confocal image illustrating *Fusobacterium* morphology in FFPE tissue sections (Fig. 5B). The same pan-*Fusobacterium* antiserum and *F. nucleatum*-specific antibody (ANT0084) were applied to the sections, together with DAPI nuclear staining. A similar pattern of staining was observed in the tissue as in cell culture, with *F. nucleatum*-positive regions displaying overlapping signals from both antibodies. The pan-*Fusobacterium* antiserum produced a clear positive signal in infected regions but also exhibited weak background staining in non-infected tissue, consistent with the observations in cell culture.

## DISCUSSION

With growing interest in tumor-associated microbiomes, WGS interpretation remains vulnerable because non-human reads constitute a small minority and taxonomic profiling tools can misclassify human reads as bacterial. To address this, we developed a workflow that prioritizes high specificity and computational efficiency, carefully excluding human reads to minimize errors in subsequent steps. We implemented a pre-screen-then-align strategy: first applying rapid k-mer-based host depletion, then performing quality and contaminant cleanup, and ultimately executing sequential alignments to complementary human references to eliminate residual host signal.

Kraken2 pre-screening against a curated human k-mer database efficiently eliminates the overwhelming bulk of human sequences and reduces data volume for all downstream operations. Because Kraken2 relies on exact k-mer matches, adapter sequence contributes no informative k-mers and typically has negligible impact at this stage. Any minor loss of k-mer informativeness caused by adapter-rich reads is corrected by the subsequent trimming and alignment stages. We therefore trim and quality-filter after pre-screening, collapse duplicate pairs, and remove vector sequences to improve mapping capability precisely where aligners are most sensitive: adapters, low-quality tails, and technical contaminants. Residual host signal is then removed with a multi-aligner, multi-reference strategy that retains only read pairs unmapped at each stage.

The three-step alignment design in HumanFilt was adopted as a pragmatic strategy to maximize host read removal, rather than to imply strong intrinsic complementarity between specific aligners and reference assemblies. Because alignment tools differ in how they search for and score matches, they may detect overlapping but not identical sets of host reads. The final alignment step still contributed additional host read removal after BWA-MEM and Bowtie2. In the HPRC-simulated data set, 53,455 reads (6.0% of all aligner-removed reads) were removed only at the final Minimap2/HPRC step. However, because these reads were simulated from the HPRC reference, this finding is better interpreted as support for including an additional pangenome-based alignment stage than as evidence of a specific advantage of Minimap2 itself. The practical cost of retaining all three steps was minimal, since Kraken2 pre-screening reduced the data set to fewer than 0.15% of the original reads before alignment. We therefore used a fixed sequential workflow to ensure applicability across data sets, since host contamination may vary across tumor types and parameter optimization is unlikely to be practical for large tumor WGS data sets. This design is broadly consistent with Bush et al. (13), who reported that, in short-read data sets, combining methods improved human-read detection and that a two-stage Bowtie2-plus-SNAP strategy was among the best-performing approaches. Although Kraken2 removed the vast majority of host-derived reads, the subsequent alignment-based filtering steps remained important, still removing millions of sequences per sample despite accounting for only a small fraction of total reads. This highlights the importance of stringent filtering in low bacterial biomass data sets, where even modest residual host contamination can distort microbial profiles.

The simulation analyses further support the interpretation that HumanFilt achieves high specificity without substantial false-negative loss of true microbial signal. In the pure *F. nucleatum* data set, the pipeline retained essentially all authentic microbial reads, and PathSeq-classified *Fusobacterium* counts increased slightly after filtering, likely because removal of competing human sequences improved assignment of previously ambiguous reads. In mixed human-microbial data sets, HumanFilt removed the overwhelming majority of host reads while preserving more than 99.7% of *Fusobacterium* signal across all reference backgrounds tested. These results suggest that HumanFilt does not systematically remove genuine *Fusobacterium* reads. Instead, the losses observed in tumor samples are likely to reflect removal of mutated cancer-derived reads that were misclassified by PathSeq as bacterial.

The critical importance of rigorous host read removal in tumor WGS microbiome studies has been powerfully demonstrated by Gihawi et al. (5), who showed that insufficient human-read depletion led to false-positive microbial detections inflated by up to four orders of magnitude, effectively invalidating findings from a large-scale cancer microbiome data set and the subsequent studies built upon it. Our work directly addresses this concern by developing and evaluating a stringent multi-step pipeline and contextualizing its performance within the landscape of existing host depletion approaches.

To contextualize HumanFilt, we performed a direct comparison against two published host removal tools:

Deacon (11) and NoHuman (10). Deacon is a minimizer-based sequence filter that queries a human pangenome index to rapidly classify and discard human-derived reads, operating at high throughput with low computational overhead. NoHuman similarly employs k-mer-based classification against a pangenome reference for fast host depletion. Cleanifier, a further pangenome-aware tool employing gapped k-mer classification against a human pangenome index, was not benchmarked as it operates on the same underlying principle.

HumanFilt achieved the most stringent host read removal across all tumor samples, retaining a mean of 0.06% of the original reads (range: 0.02%–0.09%) after filtering, compared to 0.4% (0.17%–0.91%) for Deacon and 0.11% (0.04%–0.19%) for NoHuman (Table S7; Fig. 3A). These results confirm that HumanFilt’s sequential multi-aligner strategy removes a substantially greater proportion of human-derived reads than single-step k-mer-based tools, consistent with the recommendation of Bush et al. (13) that sequential use of complementary alignment-based methods maximizes host read removal performance.

To evaluate tool specificity, we tracked *Fusobacterium* read counts before and after host depletion across all tumor samples (Table S8; Fig. 3B). Deacon and NoHuman preserved the vast majority of *Fusobacterium* reads, with mean off-target removal rates of 1.6% (range: 0.08%–7.02%) and 2.3% (range: 0.29%–9.08%), respectively. In contrast, HumanFilt removed a higher proportion of PathSeq-classified *Fusobacterium* reads, with a mean loss of 16.0% (range: 6.44%–36.06%). Importantly, simulation controls confirmed that HumanFilt does not remove true *Fusobacterium* reads under clean conditions, ruling out tool-level bias as the cause. The off-target loss observed in tumor samples is therefore most consistent with somatically mutated human reads being misclassified as *Fusobacterium* by PathSeq and then removed by HumanFilt’s more exhaustive alignment-based filtering, whereas a proportion of these reads may be retained by Deacon and NoHuman.

This highlights the central trade-off in host depletion for tumor microbiome studies: methods that maximize removal of host contamination may also reduce ambiguous microbial signal, whereas faster k-mer-based tools may preserve more reads at the cost of retaining more host-derived background.

These findings highlight a fundamental challenge in tumor microbiome research: the high somatic mutation burden characteristic of cancer genomes creates sequence overlap between host and microbial reads that k-mer-based tools cannot reliably resolve. The choice of host depletion pipeline should therefore be guided by study context. HumanFilt is recommended where maximal host read removal is the primary objective and microbial targets are robust to aggressive filtering.

Several limitations of this study should be acknowledged. First, the cohort consists of 10 colorectal cancer cases, which limits statistical power and precludes generalization of quantitative findings. Second, all samples derive from a single cancer type, and it remains to be established whether the degree of human-read contamination and fusobacterial false-positive inflation observed here is representative of other tumor types. Third, the immunohistochemical validation was performed on a subset of cases and serves as a directional indicator only, not a quantitative ground truth. These findings require validation in larger prospective cohorts.

Our immunohistochemical staining showed general consistency with WGS data, providing robust spatial and morphological confirmation of bacterial species identified by sequencing. Recent studies suggest that *F. animalis* may play a more aggressive role in colorectal cancer progression compared to *F. nucleatum* (14, 35), so calling subspecies correctly matters. Our findings emphasize the importance of careful removal of human contamination and accurate quantification of bacterial reads in WGS data for reliable detection and differentiation of *Fusobacterium* subspecies in colorectal cancer.

## Supplementary Material

Reviewer comments

## Data Availability

The HumanFilt source code is available on GitHub at https://github.com/jprehn-lab/humanfilt. The version of the HumanFilt source code and analysis scripts used for the analyses reported in this article has been archived in Zenodo at https://doi.org/10.5281/zenodo.20597190. The custom databases and supporting files associated with this study are available in Zenodo at https://doi.org/10.5281/zenodo.17020481. The Reynolds et al. (15) raw sequencing data are not publicly available due to legislative restrictions. Access to these data may be requested from the corresponding author of the Reynolds et al. publication, subject to national GDPR guidelines and institutional ethical approval. Samples from this data set are referred to in the article using anonymized case identifiers. No new human sequencing data were generated as part of this study.

## SUPPLEMENTAL MATERIAL

Supplemental tables msystems.00550-26-s0001.xlsx Tables S0 to S11.

OPEN PEER REVIEW reviewer-comments.pdf An accounting of the reviewer comments and feedback.

ASM does not own the copyrights to Supplemental Material that may be linked to, or accessed through, an article. The authors have granted ASM a non-exclusive, world-wide license to publish the Supplemental Material files. Please contact the corresponding author directly for reuse.

## References

[1] Geller LT, Barzily-Rokni M, Danino T, Jonas OH, Shental N, Nejman D, Gavert N, Zwang Y, Cooper ZA, Shee K, et al.. 2017. Potential role of intratumor bacteria in mediating tumor resistance to the chemotherapeutic drug gemcitabine. Science 357:1156–1160. doi:10.1126/science.aah5043 28912244 PMC5727343

[2] Peng F, Hu M, Su Z, Hu L, Guo L, Yang K. 2024. Intratumoral microbiota as a target for advanced cancer therapeutics. Adv Mater 36:e2405331. doi:10.1002/adma.202405331 39054925

[3] Poore Gregory D, Kopylova E, Zhu Q, Carpenter C, Fraraccio S, Wandro S, Kosciolek T, Janssen S, Metcalf J, Song SJ, Kanbar J, Miller-Montgomery S, Heaton R, Mckay R, Patel SP, Swafford AD, Knight R. 2020. Microbiome analyses of blood and tissues suggest cancer diagnostic approach. Nature 579:567–574. doi:10.1038/s41586-020-2095-1 32214244 PMC7500457

[4] Poore GD. 2020. Retraction note: microbiome analyses of blood and tissues suggest cancer diagnostic approach. Nature 631:567–574. doi:10.1038/S41586-024-07656-X 38926587

[5] Gihawi A, Ge Y, Lu J, Puiu D, Xu A, Cooper CS, Brewer DS, Pertea M, Salzberg SL. 2023. Major data analysis errors invalidate cancer microbiome findings. mBio 14:e01607-23. doi:10.1128/mbio.01607-23 37811944 PMC10653788

[6] Lu J, Salzberg SL. 2018. Removing contaminants from databases of draft genomes. PLoS Comput Biol 14:e1006277. doi:10.1371/journal.pcbi.1006277 29939994 PMC6034898

[7] Goig GA, Blanco S, Garcia-Basteiro AL, Comas I. 2020. Contaminant DNA in bacteriARal sequencing experiments is a major source of false genetic variability. BMC Biol 18:1–15. doi:10.1186/S12915-020-0748-Z 32122347 PMC7053099

[8] Gihawi A, Wood HM, Clark J, O’Grady J, Eeles RA, Wedge DC, Jakobsdottir GM, Magiorkinis G, Schache AG, Masterson L, Lechner M, Fenton TR, Jones TM, Flanagan AM, De Noon S, Rubinsteyn A, Hurst R, Cooper CS, Brewer DS. 2025. The landscape of microbial associations in human cancer. Sci Transl Med 17:6166. doi:10.1126/scitranslmed.ads6166 40901921

[9] Ge Y, Lu J, Puiu D, Revsine M, Salzberg SL. 2025. Comprehensive analysis of microbial content in whole-genome sequencing samples from The Cancer Genome Atlas project. Sci Transl Med 17:6335. doi:10.1126/scitranslmed.ads6335 PMC12821378 40901923

[10] Hall MB, Coin LJM. 2024. Pangenome databases improve host removal and mycobacteria classification from clinical metagenomic data. Gigascience 13:1–10. doi:10.1093/gigascience/giae010 PMC10993716 38573185

[11] Constantinides B, Lees J, Crook DW. 2025. Deacon: fast sequence filtering and contaminant depletion. Bioinformatics. doi:10.1101/2025.06.09.658732

[12] Zentgraf J, Schmitz JE, Rahmann S. 2026. Cleanifier: contamination removal from microbial sequences using spaced seeds of a human pangenome index. Bioinformatics 42:1. doi:10.1093/bioinformatics/btaf632 PMC12758600 41252442

[13] Bush SJ, Connor TR, Peto TEA, Crook DW, Walker AS. 2020. Evaluation of methods for detecting human reads in microbial sequencing datasets. Microb Genom 6:5–18. doi:10.1099/mgen.0.000393 PMC7478626 32558637

[14] Zepeda-Rivera M, Minot SS, Bouzek H, Wu H, Blanco-Míguez A, Manghi P, Jones DS, LaCourse KD, Wu Y, McMahon EF, Park S-N, Lim YK, Kempchinsky AG, Willis AD, Cotton SL, Yost SC, Sicinska E, Kook J-K, Dewhirst FE, Segata N, Bullman S, Johnston CD. 2024. A distinct *Fusobacterium nucleatum* clade dominates the colorectal cancer niche. Nature 628:424–432. doi:10.1038/s41586-024-07182-w 38509359 PMC11006615

[15] Reynolds IS, Thomas V, O’Connell E, Fichtner M, McNamara DA, Kay EW, Prehn JHM, Burke JP, Furney SJ. 2020. Mucinous adenocarcinoma of the rectum: a whole genome sequencing study. Front Oncol 10:1682. doi:10.3389/fonc.2020.01682 32984045 PMC7479243

[16] Wood DE, Lu J, Langmead B. 2019. Improved metagenomic analysis with Kraken 2. Genome Biol 20:257. doi:10.1186/s13059-019-1891-0 31779668 PMC6883579

[17] Schneider VA, Graves-Lindsay T, Howe K, Bouk N, Chen H-C, Kitts PA, Murphy TD, Pruitt KD, Thibaud-Nissen F, Albracht D, et al.. 2017. Evaluation of GRCh38 and de novo haploid genome assemblies demonstrates the enduring quality of the reference assembly. Genome Res 27:849–864. doi:10.1101/gr.213611.116 28396521 PMC5411779

[18] Xu H, Luo X, Qian J, Pang X, Song J, Qian G, Chen J, Chen S. 2012. FastUniq: a fast de novo duplicates removal tool for paired short reads. PLoS One 7:e52249. doi:10.1371/journal.pone.0052249 23284954 PMC3527383

[19] Bushnell B. 2014. BBMap: a fast, accurate, splice-aware aligner. Available from: https://github.com/bbushnell/BBTools

[20] Li H. 2013. Aligning sequence reads, clone sequences and assembly contigs with BWA-MEM. Available from: http://github.com/lh3/bwa. Retrieved 28 Oct 2025.

[21] Nurk S, Koren S, Rhie A, Rautiainen M, Bzikadze AV, Mikheenko A, Vollger MR, Altemose N, Uralsky L, Gershman A, et al.. 2022. The complete sequence of a human genome. Science 376:44–53. doi:10.1126/science.abj6987 35357919 PMC9186530

[22] Langmead B, Salzberg SL. 2012. Fast gapped-read alignment with Bowtie 2. Nat Methods 9:357–359. doi:10.1038/nmeth.1923 22388286 PMC3322381

[23] Liao W-W, Asri M, Ebler J, Doerr D, Haukness M, Hickey G, Lu S, Lucas JK, Monlong J, Abel HJ, et al.. 2023. A draft human pangenome reference. Nature 617:312–324. doi:10.1038/s41586-023-05896-x 37165242 PMC10172123

[24] Li H. 2018. Minimap2: pairwise alignment for nucleotide sequences. Bioinformatics 34:3094–3100. doi:10.1093/bioinformatics/bty191 29750242 PMC6137996

[25] Kostic AD, Ojesina AI, Pedamallu CS, Jung J, Verhaak RGW, Getz G, Meyerson M. 2011. PathSeq: software to identify or discover microbes by deep sequencing of human tissue. Nat Biotechnol 29:393–396. doi:10.1038/nbt.1868 21552235 PMC3523678

[26] Blanco-Míguez A, Beghini F, Cumbo F, McIver LJ, Thompson KN, Zolfo M, Manghi P, Dubois L, Huang KD, Thomas AM, et al.. 2023. Extending and improving metagenomic taxonomic profiling with uncharacterized species using MetaPhlAn 4. Nat Biotechnol 41:1633–1644. doi:10.1038/s41587-023-01688-w 36823356 PMC10635831

[27] Huang W, Li L, Myers JR, Marth GT. 2012. ART: a next-generation sequencing read simulator. Bioinformatics 28:593–594. doi:10.1093/bioinformatics/btr708 22199392 PMC3278762

[28] Shen W, Le S, Li Y, Hu F. 2016. SeqKit: a cross-platform and ultrafast toolkit for FASTA/Q file manipulation. PLoS One 11:e0163962. doi:10.1371/journal.pone.0163962 27706213 PMC5051824

[29] Casasanta MA, Yoo CC, Udayasuryan B, Sanders BE, Umaña A, Zhang Y, Peng H, Duncan AJ, Wang Y, Li L, Verbridge SS, Slade DJ. 2020. *Fusobacterium nucleatum* host-cell binding and invasion induces IL-8 and CXCL1 secretion that drives colorectal cancer cell migration. Sci Signal 13:eaba9157. doi:10.1126/scisignal.aba9157 32694172 PMC7454160

[30] Duggan WP, Salvucci M, Kisakol B, Lindner AU, Reynolds IS, Dussmann H, Fay J, O’Grady T, Longley DB, Ginty F, Mc Donough E, Slade DJ, Burke JP, Prehn JHM. 2023. Increased *Fusobacterium* tumoural abundance affects immunogenicity in mucinous colorectal cancer and may be associated with improved clinical outcome. J Mol Med (Berlin) 101:829–841. doi:10.1007/s00109-023-02324-5 37171483 PMC10300184

[31] Dussmann H, Maguire B, Burke C, Raman A, Burke J, Gallagher WM, Prehn JH. 2025. Abstract 6531: high resolution analysis of Fusobacterium infection in colorectal cancer. Cancer Res 85:6531–6531. doi:10.1158/1538-7445.AM2025-6531

[32] Rubinstein MR, Wang X, Liu W, Hao Y, Cai G, Han YW. 2013. *Fusobacterium* nucleatum promotes colorectal carcinogenesis by modulating E-cadherin/β-catenin signaling via its FadA adhesin. Cell Host Microbe 14:195–206. doi:10.1016/j.chom.2013.07.012 23954158 PMC3770529

[33] Krueger F. 2025. Trim Galore. Available from: https://www.bioinformatics.babraham.ac.uk/projects/trim_galore/. Retrieved 24 October 2025.

[34] Sepich-Poore GD, McDonald D, Kopylova E, Guccione C, Zhu Q, Austin G, Carpenter C, Fraraccio S, Wandro S, Kosciolek T, Janssen S, Metcalf JL, Song SJ, Kanbar J, Miller-Montgomery S, Heaton R, Mckay R, Patel SP, Swafford AD, Korem T, Knight R. 2024. Robustness of cancer microbiome signals over a broad range of methodological variation. Oncogene 43:1127–1148. doi:10.1038/s41388-024-02974-w 38396294 PMC10997506

[35] Ye X, Wang R, Bhattacharya R, Boulbes DR, Fan F, Xia L, Adoni H, Ajami NJ, Wong MC, Smith DP, Petrosino JF, Venable S, Qiao W, Baladandayuthapani V, Maru D, Ellis LM. 2017. *Fusobacterium* nucleatum subspecies animalis influences proinflammatory cytokine expression and monocyte activation in human colorectal tumors. Cancer Prev Res (Philadelphia) 10:398–409. doi:10.1158/1940-6207.CAPR-16-0178 28483840

