# Supplementary material for: HumanFilt: a multi-reference host depletion pipeline improves Fusobacterium detection accuracy in tumor WGS data sets
Source: mSystems. 2026 Jul 14;11(8):e00550-26. doi: 10.1128/msystems.00550-26 (PMC13483447; doi:10.1128/msystems.00550-26)
Supplement: Supplementary file 1 [file reviewer-comments.pdf]

AMERICAN SOCIETY FOR MICROBIOLOGY | mSystems®

HumanFilt: a multi-reference host depletion pipeline improves *Fusobacterium* detection accuracy in tumour WGS datasets.

Mariia Frolova, Barry Maguire, Heiko Duessmann, Menatallah Rayan, Caoimbhe Burke, Hesham Korashy, Arman Raman, William Gallagher, John Burke, Simon Furney, and Jochen Prehn

Corresponding Author(s): Jochen Prehn, Royal College of Surgeons in Ireland

Review Timeline:

Submission Date:	April 29, 2026
Editorial Decision:	June 3, 2026
Revision Received:	June 15, 2026
Accepted:	June 18, 2026

Editor: Germán Bonilla-Rosso

Reviewer(s): The reviewers have opted to remain anonymous.

Transaction Report:

(Note: With the exception of the correction of typographical or spelling errors that could be a source of ambiguity, letters and reports are not edited. The original formatting of letters and referee reports may not be reflected in this compilation.)

DOI: https://doi.org/10.1128/msystems.00550-26

Re: mSystems00550-26 (HumanFilt: a multi-reference host depletion pipeline improves *Fusobacterium* detection accuracy in tumour WGS datasets.)

Dear Prof. Jochen H. M. Prehn:

Thank you for the privilege of reviewing your work. Below you will find my comments, instructions from the mSystems editorial office, and the reviewer comments.

Thank you for your resubmission. The reviewers are satisfied with how you've addressed most of their concerns, and have only minor comments. However there are some minor issues regarding data availability that must be addressed:

1. As per ASM policy, please add a "Data Availability" paragraph (https://journals.asm.org/open-data-policy).

2. Please add a Zenodo link that is easily followed, as it cannot be visited nor searched in Zenodo in its current version (DOI 10.5281/zenodo.17020481). Perhaps using the DOI address (https://doi.org/10.5281/zenodo.17020481) would be better.

3. Thank you for linking to your GitHub repository. However, to ensure that the code used for the analysis in the manuscript remains available, I would request that you upload a version of your code to Zenodo as well.

Thank you, and I would not expect to send your resubmission to another round of reviews.

Please return the manuscript within 60 days; if you cannot complete the modification within this time period, please contact me. If you do not wish to modify the manuscript and prefer to submit it to another journal, notify me immediately so that the manuscript may be formally withdrawn from consideration by mSystems.

Revision Guidelines
To submit your modified manuscript, log into the submission site at https://msystems.msubmit.net/cgi-bin/main.plex. Go to Author Tasks and click the appropriate manuscript title to begin. The information you entered when you first submitted the paper will be displayed; update this as necessary. Note the following requirements:

• Upload point-by-point responses to the issues raised by the reviewers in a file named "Response to Reviewers," NOT in your cover letter.
• Upload a compare copy of the manuscript (without figures) as a "Marked-Up Manuscript" file.
• Upload a clean .DOC/.DOCX version of the revised manuscript and remove the previous version.
• Each figure must be uploaded as a separate, editable, high-resolution file (TIFF or EPS preferred), and any multipanel figures must be assembled into one file.
• Any supplemental material intended for posting by ASM should be uploaded with their legends separate from the main manuscript. You can combine all supplemental material into one file (preferred) or split it into a maximum of 10 files with all associated legends included.

For complete guidelines on revision requirements, see our Submission and Review Process webpage. Submission of a paper that does not conform to guidelines may delay acceptance of your manuscript.

Data availability: ASM policy requires that data be available to the public upon online posting of the article, so please verify all links to sequence records, if present, and make sure that each number retrieves the full record of the data. If a new accession number is not linked or a link is broken, provide mSystems production staff with the correct URL for the record. If the accession numbers for new data are not publicly accessible before the expected online posting of the article, publication may be delayed; please contact production staff (mSystems@asmusa.org) immediately with the expected release date.

Publication Fees: For information on publication fees and which article types are subject to charges, visit our website. If your manuscript is accepted for publication and any fees apply, you will be contacted separately about payment during the production process; please follow the instructions in that e-mail. Arrangements for payment must be made before your article is published.

ASM Membership: Corresponding authors may join or renew ASM membership to obtain discounts on publication fees. Need to upgrade your membership level? Please contact Customer Service at Service@asmusa.org.

The ASM Journals program strives for constant improvement in our submission and publication process. Please tell us how we can improve your experience by taking this quick Author Survey.

Thank you for submitting your paper to mSystems.

Sincerely,

Germán Bonilla-Rosso
Editor
mSystems

Reviewer #1 (Comments for the Author):

I thank the authors for making their extensive revisions, which have addressed all of my previous comments. I had only a few relatively minor comments on the revised draft, mostly correcting very minor errors. Note that line numbers indicated below refer to the marked up version of the manuscript:

General comment - there are still quite a lot of uses of "abundance" throughout the revised document (including in figure labels/headings) that should be "proportional abundance"

Line 113 - should be "are listed"?

Line 129 - please add a reference for "ART (version MountRainier)", in order to give appropriate credit to the software developers

Line 162 - "F. nucleatum subsp. nucleatum 25586" should be "F. nucleatum subsp. nucleatum ATCC 25586"

Lines 254 and 323 - "Fusobacterium" should be in italics

Line 268 - "remove" should be "removed"?

Reviewer #3 (Comments for the Author):

I thank the authors for responding to reviewer comments and for addressing my concerns in the revised manuscript, which I appreciate involved some additional work. The authors may wish to publicly archive their software using a service such as Zenodo to ensure its future availability, if not already done so.

Response to Reviewers

Manuscript ID: mSystems00550-26

Title: HumanFilt: a multi-reference host depletion pipeline improves Fusobacterium detection accuracy in tumour WGS datasets

Dear Dr. Bonilla-Rosso and Reviewers,

We thank the editor and reviewers for their careful assessment of our revised manuscript and for their positive and constructive comments. We are pleased that the reviewers were satisfied with the major revisions. We have addressed all remaining editorial and reviewer comments in the revised manuscript. The changes include adding a dedicated Data Availability statement, updating Zenodo links to full DOI URLs, archiving the HumanFilt source code and analysis scripts on Zenodo, and correcting the minor textual and formatting issues noted by the reviewers.

A point-by-point response is provided below.

Response to Editor

1) As per ASM policy, please add a "Data Availability" paragraph.

We have added a dedicated Data Availability paragraph to the revised manuscript. This paragraph includes links to the HumanFilt GitHub repository, the archived Zenodo record for the HumanFilt source code and analysis scripts, and the Zenodo record containing the custom databases and supporting files. It also clarifies the availability status of the Reynolds et al. raw sequencing data.

2) Please add a Zenodo link that is easily followed, as it cannot be visited nor searched in Zenodo in its current version (DOI 10.5281/zenodo.17020481). Perhaps using the DOI address (https://doi.org/10.5281/zenodo.17020481) would be better.

We have updated the Zenodo citation to use a full DOI URL. The relevant Zenodo record is now provided as an easily followed DOI link in the Data Availability statement.

3) Thank you for linking to your GitHub repository. However, to ensure that the code used for the analysis in the manuscript remains available, I would request that you upload a version of your code to Zenodo as well.

We have archived the HumanFilt source code and analysis scripts used for the analyses reported in this manuscript in Zenodo. The Zenodo DOI has been added to the Data Availability statement: https://doi.org/10.5281/zenodo.20597190 .

Response to Reviewer #1

1) There are still quite a lot of uses of "abundance" throughout the revised document, including in figure labels/headings, that should be "proportional abundance".

We thank the reviewer for this helpful clarification. We have revised the manuscript to replace vague uses of "abundance" with more specific terminology where appropriate. Where values are expressed as proportions or percentages, we now use "proportional abundance" or retain

"relative abundance" where this term was already used. Where values are expressed as reads per million reads (RPM), we now use "RPM values", "normalised read abundance", or "normalised read counts" as appropriate, to avoid conflating RPM-based measurements with proportional abundance. Figure labels, headings, and captions were revised accordingly where appropriate.

2) *line 113:* Should be "are listed"?

We have corrected this wording to "are listed".

3) line 129: Please add a reference for "ART (version MountRainier)", in order to give appropriate credit to the software developers.

We have added the appropriate reference for ART and clarified the software version as ART_Illumina version 2.5.8 (June 6, 2016).

3) line 162: "F. nucleatum subsp. nucleatum 25586" should be "F. nucleatum subsp. nucleatum ATCC 25586".

We have corrected this to "F. nucleatum subsp. nucleatum ATCC 25586".

3) lines 254 and 323: "Fusobacterium" should be in italics.

We have italicised *Fusobacterium* at the indicated locations.

4) line 268: "remove" should be "removed"?

We have corrected this wording to "removed".

Response to Reviewer #3

1) I thank the authors for responding to reviewer comments and for addressing my concerns in the revised manuscript, which I appreciate involved some additional work. The authors may wish to publicly archive their software using a service such as Zenodo to ensure its future availability, if not already done so.

We thank the reviewer for this helpful suggestion. The HumanFilt source code and the analysis scripts used for the manuscript have now been publicly archived on Zenodo to ensure long-term availability. The archived code record is available at https://doi.org/10.5281/zenodo.20597190 and has been added to the Data Availability statement.

Re: mSystems00550-26R1 (HumanFilt: a multi-reference host depletion pipeline improves *Fusobacterium* detection accuracy in tumour WGS datasets.)

Dear Prof. Jochen H. M. Prehn:

Thank you for answering all issues raised by the reviewers and incorporated a Data Availability paragraph. Your manuscript has been accepted, and I am forwarding it to the ASM production staff for publication. Your paper will first be checked to make sure all elements meet the technical requirements. ASM staff will contact you if anything needs to be revised before copyediting and production can begin. Otherwise, you will be notified when your proofs are ready to be viewed.

Data Availability: ASM policy requires that data be available to the public upon online posting of the article, so please verify all links to sequence records, if present, and make sure that each number retrieves the full record of the data. If a new accession number is not linked or a link is broken, provide production staff with the correct URL for the record. If the accession numbers for new data are not publicly accessible before the expected online posting of the article, publication may be delayed; please contact ASM production staff immediately with the expected release date.

Publication Fees: For information on publication fees and which article types have charges, please visit our website. We have partnered with Copyright Clearance Center (CCC) to collect author charges. If fees apply to your paper, you will receive a message from no-reply@copyright.com with further instructions. For questions related to paying charges through RightsLink, please contact CCC at ASM_Support@copyright.com or toll free at +1-877-622-5543. CCC makes every attempt to respond to all emails within 24 hours.

ASM Membership: Corresponding authors may join or renew ASM membership to obtain discounts on publication fees. Need to upgrade your membership level? Please contact Customer Service at Service@asmusa.org.

Embargo Policy: A press release may be issued as soon as the manuscript is posted on the mSystems Latest Articles webpage. The corresponding author will receive an email with the subject line "ASM Journals Author Services Notification" when the article is available online.

Cover Image Submissions: If you would like to submit a potential Cover Image, please email a file and a short legend to msystems@asmusa.org. Please note that we can only consider images that (i) the authors created or own and (ii) have not been previously published. By submitting, you agree that the image can be used under the same terms as the published article. Image File requirements: TIF/EPS, 7.5 inches wide by 8.25 inches tall (at least 2,250 pixels wide by 2,475 pixels tall), minimum 300 dpi resolution (600 dpi preferred), RGB, and no figure elements, e.g., arrows or panel labels. The legend should be a short description of the image, 1-2 sentences recommended. Please download and use this interactive template in Adobe to ensure that your proposed cover image meets our size requirements (https://journals.asm.org/pb-assets/pdf-text-excel-files/ASM-Interactive-Sizing-Cover-Template-1715689791.pdf).

Thank you for submitting your paper to mSystems.

Sincerely,
Germán Bonilla-Rosso
Editor
mSystems
